# MicroRNAs Mediated Plant Responses to Salt Stress

**DOI:** 10.3390/cells11182806

**Published:** 2022-09-08

**Authors:** Waqar Islam, Abdul Waheed, Hassan Naveed, Fanjiang Zeng

**Affiliations:** 1 Xinjiang Key Laboratory of Desert Plant Roots Ecology and Vegetation Restoration, Xinjiang Institute of Ecology and Geography, Chinese Academy of Sciences, Urumqi 830011, China; 2 State Key Laboratory of Desert and Oasis Ecology, Xinjiang Institute of Ecology and Geography, Chinese Academy of Sciences, Urumqi 830011, China; 3 Cele National Station of Observation and Research for Desert-Grassland Ecosystems, Cele 848300, China; 4 University of Chinese Academy of Sciences, Beijing 100049, China; 5 College of Life Sciences, Leshan Normal University, Chengdu 614004, China

**Keywords:** abiotic stresses, environmental constraints, genetic regulations, salinity stress, arid environment

## Abstract

One of the most damaging issues to cultivatable land is soil salinity. While salt stress influences plant growth and yields at low to moderate levels, severe salt stress is harmful to plant growth. Mineral shortages and toxicities frequently exacerbate the problem of salinity. The growth of many plants is quantitatively reduced by various levels of salt stress depending on the stage of development and duration of stress. Plants have developed various mechanisms to withstand salt stress. One of the key strategies is the utilization of microRNAs (miRNAs) that can influence gene regulation at the post-transcriptional stage under different environmental conditions, including salinity. Here, we have reviewed the miRNA-mediated adaptations of various plant species to salt stress and other abiotic variables. Moreover, salt responsive (SR)-miRNAs, their targets, and corresponding pathways have also been discussed. The review article concludes by suggesting that the utilization of miRNAs may be a vital strategy to generate salt tolerant crops ensuring food security in the future.

## 1. Introduction

Salinity is characterized by the accumulation of large levels of soluble salts in the soil, resulting in a high salt concentration that is harmful to plant growth [1]. Hyper-ionic and hyper-osmotic stress are caused by high salinity, resulting in physiological drought conditions that can harm plant species [2]. Prolonged saline conditions result in the production of reactive oxygen species (ROS), including an increase in mono-oxygen, superoxide, hydroxyl radicals, and hydrogen peroxide [2]. Salinity-induced ROS causes oxidative damage to cellular components such as proteins, lipids, and DNA and disrupts key plant biological activities [3]. A reversible slowdown in metabolism and growth or irreversible cell death could occur due to tissue injuries. Such conditions make soil salinity a crucial stress among abiotic factors. 

Salinization arises because of an imbalance in the water cycle when water runs out on irrigated land and salt accumulates because of inadequate drainage [4]. With the expansion of irrigation networks, salinity is also increasing. Salinization is estimated to affect nearly 20% of total irrigated land. That percentage is likely to climb to 30% in the following two and a half decades and to around 50% by the middle of the 21st century [5]. Saline soils have caused significant agronomic problems in Asia, Africa, and South America’s dry and semi-arid regions, with around 20% of cultivated land facing salinity stress [6]. 

Plants’ responses to salinity have complicated physiological characteristics, embedding several changes in metabolic processes and implications of gene networks [7]. MicroRNAs (miRNAs) are 20–22 nucleotide regulatory RNAs encoded by endogenous *miR* genes in plants [8]. Their primary transcripts generate precursor RNAs with a partially double-stranded stem-loop structure, which *Dicer-like (DCL)* proteins convert into mature miRNAs. In the miRNA biogenesis pathway, RNA polymerase II (Pol II) transcribes primordial miRNAs (pri-miRNAs) from nuclear-encoded *miR* genes, resulting in precursor transcripts with a distinctive hairpin structure [9]. *DCL1* converts pri-miRNA into pre-miRNA with the help of the *HYPONASTIC LEAVES 1 (HYL1)* and *SERRATE (SE)* proteins [10,11]. miRNA duplexes are formed by the de novo synthesis of the pre-miRNA hairpin precursor regulated by *DCL1*, *HYL1*, and *SE*. *HASTY (HST1)*, an exportin protein, is sent into the cytoplasm after methylation by *Hua enhancer 1 (HEN1)* [12,13]. RNA-induced silencing complex’s (RISC’s) catalytic component, *Argonaute (AGO)* protein, binds one strand of the duplex (miRNA) in the cytoplasm and uses sequence complementarity to direct RISC to cognate target transcripts [14]. miRNAs influence gene expression by creating epigenetic changes like DNA and histone methylation and influencing post-transcriptional targets [15,16]. Several excellent reviews have recently been published on the biogenesis of miRNAs in plants [10,11,13,17,18,19,20,21].

In eukaryotes, miRNAs influence gene expression after the occurrence of transcription [22]. miRNA strands are normally destroyed, but they can also be transformed into functional guide strands that play regulatory roles in plants [23]. In addition to their role in plant growth and developmental processes, miRNAs regulate plant responses to biotic and abiotic stress conditions [24,25,26]. Many salinity-responsive miRNAs and their targets in plants have been found. Some of the miRNAs, their target genes, and their functions are given in Table 1. Keeping in view the regulatory role of miRNAs during saline conditions, this review focused on new insights about plant miRNAs responsive to salt stress and miRNA-associated regulatory networks that trigger molecular events in plants under such conditions. Correspondingly, this review also highlighted the recent findings about SR-miRNAs, their target genes, and corresponding pathways.

## 2. Abiotic Stress Responsive miRNAs in Plants

When confronted with environmental stresses, some miRNAs exhibit altered regulations. Nutrient imbalance [27,28], drought [29,30,31], salinity [32,33,34], cold [35,36,37], heat or high temperatures [38,39,40], ultraviolet (UV)-B/C rays [41,42] result in altered expressions of various miRNA families. miR398 was the first miRNA to be directly linked to environmental stress tolerance in plants. miR398 specifically targets two genes encoding Copper/Zinc (Cu/Zn) and superoxide dismutases (SODs) in Arabidopsis; CSD1 (cytosolic) and CSD2 (extracellular) [43]. Sunkar et al. [44] discovered that down-regulating miR398 increased oxidative stress tolerance in transgenic lines than in wild-type (WT) plants. 

The expression levels of 117 miRNAs in Arabidopsis were determined using microarray technology and miRNA chips under salt and other abiotic stress conditions [45]. Examining how their promoter regions affect cis-regulation and assessing their expression patterns verified the findings of more than a dozen stress-induced miRNAs [45]. A new miRNA family targeting *SOD*, *laccases (LACs)*, and *ATP sulfurylases (APS)* has been discovered in Arabidopsis [46]. Sulfate (S) deficiency increased the expression of miR395, demonstrating that miRNA expression could arise under environmental stress conditions [47]. Inorganic S assimilation is performed by the *APS*-encoding genes *(APS1*, *APS3*, *and APS4)*, which are targeted by miR395 [48]. Cold, dryness, excessive salinity, and abscisic acid (ABA) treatments, have all been found to up-regulate miR393 [49]. A response to all stressors up-regulated miR397b and miR402 [50]; however, miR319c was only up-regulated in a cold environment [51], while responses to all stressors resulted in miR398a down-regulation [52].

Abiotic stress has been shown to differentially affect miR394 expression profiles [45]. Ren et al. [53] showed that several gene-encoding transcription factors (TFs) and many enzymes were targeted by pto-miR394, whose expression increased with drought but decreased under high water conditions. These included gene-encoding *F-box* domains, *MYB (myeloblastosis)-like* DNA-binding domains, ABC transporter transmembrane regions, DNA polymerase family B, and putative methyltransferases. Several stress conditions, including high salinity, drought, and low temperatures, were used to induce stress in Arabidopsis, which resulted in the identification of 14 stress-inducible miRNAs [45]. A real-time reverse transcription–polymerase chain reaction (RT-PCR) confirmed that these stress-inducible miRNAs were differentially expressed. Three miRNAs were up-regulated during these stressful conditions, i.e., miR168, miR171, and miR396.

Many microbial and metal stress-responsive miRNAs have been uncovered in plants [23,25,26]. According to Wang et al. [54], there were 25 known and nine novel miRNAs in *Raphanus sativus* that showed differential expression under lead (Pb) stress. Several stress-related signaling and secondary metabolite pathways are primarily targeted by Pb-responsive miRNAs. In response to low nitrogen stress, Wang et al. [55] observed differential expression of some soybean miRNAs via utilization of deep sequencing (DS). The authors further discovered that the potential targets of these miRNAs were involved in different biological functions.

In two tea plant cultivars that had been treated with cold stress, Zhang et al. [56] identified 106 known miRNAs and 98 potentially novel miRNAs. The authors also uncovered 238 common targets in response to cold and control treatment. In addition, 455 and 591 genes were identified as miRNA cleavage targets in cold and control treatment groups, respectively. According to gene ontology (GO) annotations, miRNA target genes were involved in transcription, stress responses, and developmental processes. These results offered valuable insight into miRNA functions associated with cold stress in tea cultivars. There are at least 40 plant miRNA families associated with abiotic stress. These families are mostly related to salt and drought stress [55,57,58]. Candar-Cakir et al. [59] applied small RNA (sRNA) and degradome sequencing (DgS) to systematically identify tissue-specific miRNAs in multiple tomato genotypes under drought stress. The key miRNAs involved in the tomato response to drought stress included miR160, miR165, miR166, miR171, miR398, miR408, miR827, miR9472, miR9476, and miR9552. Moreover, miR169, miR172, miR393, miR5641, miR5658, and miR7997 differentially regulated the genes involved in plant hormone signal transduction pathways in all tissues regardless of genotype [59]. miRNAs do not need to be involved in plant stress adaptation, even though they are differentially regulated in response to environmental stresses.

It has been observed that some miRNAs are responsible for increasing plant tolerance to multiple environmental stresses. For example, in Bermuda grass grown under combined cold and salt stresses, out of five miRNAs (miR827-5p, PC-3p-49895, PC-5p-104176, PC-5p-56353, and PC-5p-67388), the down-regulation of three miRNAs (osa-miR160, osa-miR160a-5p, and PC-5p-131796) was observed [60]. It has been found that *Glycine max* miR169l-3p, miR5036, miR862a, and miR398a/b targeted ethylene-responsive *TF4*, *protein phosphatase 2C*, and *Cu/Zn-SOD* as a response to single and double stress phosphate starvation and salt [61]. Salinity and alkalinity treatments increased the sensitivity of rice seedlings overexpressing miR393 [62], while salt stress tolerance increased in plants overexpressing a miR393-resistant *transport inhibitor response protein 1 (TIR1)*. A higher germination rate, increased water-use efficiency, delayed senescence, and stabilized chlorophyll levels were observed in *A. thaliana* plants under saline conditions [63]. Other studies found that exogenous pri-osa-miR393a conferred enhanced drought (heat) and salt tolerance in transgenic *Agrostis stolonifera* (creeping bentgrass) plants [64]. Plants expressing the transgene had fewer but longer tillers, reduced stomatal density, denser cuticles, increased potassium uptake, and enhanced expression of small heat-shock proteins, exhibiting the plant’s ability to withstand multiple stresses can be improved via genetic incorporation of miR393.

As a result of overexpression of miR408 in Arabidopsis, salinity, cold, and oxidative stress tolerance were enhanced, but drought sensitivity and osmotic stress sensitivity were also increased [65]. *Nicotiana benthamiana* responded similarly when *Salvia miltiorrhiza* miR408 was heterologously expressed. There was reduced ROS accumulation in the plants and higher tolerance to salt stress [66]. A study showed that osa-miR408 overexpression improved drought tolerance in perennial ryegrass [67] and chickpea [68]. It is possible that decreased leaf water loss is associated with morphological changes in transgenic plants, such as curled leaves and sunken stomata. It has been demonstrated that TaemiR408, a miRNA family member in wheat *(Triticum aestivum)*, also exhibited induced expression patterns when exposed to salt stress and phosphate starvation and that its induced expression was gradually repressed when the plants reverted to normal conditions [69]. It appeared that miR408 played a crucial role in improving plant tolerance to multiple stresses caused by several abiotic factors.

Multiple stress conditions typically up-regulate miR319 via targeting *Teosinte branched 1*, *Cycloidea*, and *proliferating cell nuclear antigen binding factor (TCP/PCF)* [70]. Increasing miR319 expression increased creeping bentgrass’s tolerance for salt and drought stress [71]. Across transgenic potato plants, water retention and membrane integrity were increased under salt stress conditions, and Na^+^ accumulation was reduced [72]. osa-miR319 overexpression reduced *OsPCF5* and *OsPCF8* expression levels in rice, resulting in cold stress tolerance [73]. When considered together, the overexpression of miR319 in plants improved their tolerance to various environmental stresses. Genetically engineered tobacco plants expressing zmmiR156 were more resistant to drought and salt without compromising their architecture because their transcript levels of senescence-associated genes were reduced [74]. According to these findings, miRNAs could be useful in developing crop varieties that are more resistant to multiple stresses, thereby enhancing the productivity of agriculture.

## 3. Plant miRNAs and Salt Stress

During salt stress, numerous gene transcripts are variably regulated by miRNAs, indicating that transcription in stressed plants is tightly modulated; hence, salt stress interaction is heavily influenced by post-translational gene regulations (Table 1). There was a discrepancy between leaf and root miR398 levels of salt-stressed *Populus euphratica* as miR398 levels increased in the former while decreased in the latter. The expression of three miRNAs (miR164, miR166, and miR169) significantly altered *P. euphratica* when exposed to salt [75]. Numerous environmental factors, including salt stress, affected the expression of a diverse set of miRNAs in *P. trichocarpa* [76]. Furthermore, Phaseolus vulgaris harbored increased levels of miRNA-targeting genes implicated in Calmodulin-binding transcription activators (CAMTAs) [77]. During salt stress, the differential expression of two miR169 family members was also observed, i.e., miR169g and miR169n [78]. Upstream of miR169n, a cis-acting, ABA-responsive region was discovered, suggesting that this stress-responsive hormone regulates miR169n. The wheat leaves miR169 down-regulated via salinity expressed NF-YA (nuclear factor Y subunit A) [79]. Researchers found that salt-sensitive (SS) and salt-tolerant (ST) Zea mays cultivars showed down-regulation of miR156, miR164, miR171, miR167, and miR396, and up-regulation of miR162, miR168, miR395, and miR474 [80].

**Table 1:** Salt-responsive microRNA, their corresponding targets, and regulations in various plant species.

microRNA	Target	Plant Species	miRNA/Target Module Function	Regulations		References
				Up	Down	
miR156	Unknown	*Gossypium raimondii*	Abiotic stress tolerance			[81]
	SPLs	*Raphanus sativus*	Delays flowering; regulates leaf development, fruit ripening, vegetative and reproductive stage transitions; tillering and branching; plays key roles in embryogenesis, morphogenesis, life cycle stage transformation, and flower formation.			[82]
	SPLs	*Panicum virgatum*				[83]
	SPLs	*Arabidopsis thaliana*				[45]
	SPLs	*Malus domestica*				[84]
	POPTR_0007s01030	*Populus trichocarpa*	Unknown			[85]
	Unknown	*Medicago truncatula*	Abiotic stress tolerance			[86]
	Unknown	*Solanum lycopersicum*				[87]
	UGTs	*Hordeum spontaneum*	Increases anthocyanin synthesis, leading to enhanced antioxidative capacity.			[88]
miR157	SPLs	*P. virgatum*	Modulate leaf initiation rate			[83]
	Unknown	*G. raimondii*	Regulation of biological processes			[81]
miR159	MYBs	*Oryza sativa*	Growth and flowering, role in fruit development.			[89]
	MYBs	*P. virgatum*				[83]
	MYBs	*Nicotiana tabacum*				[90]
	MYBs	*M. truncatula*				[91]
miR160	ARFs	*G. raimondii*	Regulating plant growth and development through auxin signaling pathways			[81]
	ARFs	*R. sativus*				[82]
	ARFs	*O. sativa*				[89]
	ARFs	*Setaria italica*				[92]
	ARFs	*Triticum aestivum*				[93]
miR161	AGO	*A. thaliana*	Vital in salinity stress response			[94]
miR162	DCLs	*S. italica*	miRNA biogenesis plays a vital role in saline and drought conditions			[92]
	DCLs	*P. virgatum*				[83]
miR164	NAC	*R. sativus*	Critical role in regulating the response to salt and drought stress			[82]
	NAC	*A. thaliana*				[95]
	NAC	*P.* *euphratica*				[96]
	Pavirv00056088m	*P.* *virgatum*	Despite regulating salt stress, involvement in any other regulatory mechanisms is still unknown.			[81]
	POPTR_0007s08420	*P.* *trichocarpa*				[97]
	GRMZM2G114850	*Zea mays*				[98]
miR165	unknown	*T. aestivum*	Determining the positional fate of leaf tissues (adaxial or abaxial) and xylem differentiation in root stele tissues			[99]
	HD-ZIP	*A. thaliana*				[45]
miR166	Unknown	*G. raimondii*	Plant development processes and abiotic stresses resistance			[81]
	SPB-like	*A. thaliana*				[95]
	SPB-like	*Glycine max*				[100]
	SPB-like	*Z. mays*				[101]
miR167	Unknown	*G. raimondii*	Regulates some reproductive development processes, such as anther dehiscence, and ovule, embryonic, and seed development.			[81]
	ARF	*A. thaliana*				[45]
	ARF	*N. tabacum*				[90]
	ARF	*T. aestivum*				[93]
	ARF	*Z. mays*				[102]
miR168	AGOs	*Saccharum spp.*	Facilitates plant adaptation to K^+^-deficiency stress, influences phase transition, leaf epinasty, and fruit development			[103]
	AGOs	*A. thaliana*				[45]
	MYBs	*P. euphratica*				[84]
	AGOs	*Z. mays*				[102]
	Unknown	*G. raimondii*				[81]
	Unknown	*Vigna unguiculata*				[104]
miR169	NY-FA	*Z. mays*	Regulates tolerance to abiotic stresses in both monocots and dicots; plays a key role in nutrient uptake.			[78]
	CCAAT-binding	*A. thaliana*				[45]
	CBF HAP2-like factor	*N. tabacum*				[90]
	CCAAT-binding TF	*P. euphratica*				[84]
	CBF HAP2-like factor	*G. max*				[105]
	CCAAT-binding TF	*V. unguiculata*				[104]
miR171	Scarecrow-like TFs	*A. thaliana*	Plant growth and development			[45]
	AP2	*P. trichocarpa*				[84]
	AP2	*S. italica*				[92]
	Unknown	*S. lycopersicum*				[86]
miR172	AP2	*G. raimondii*	Regulates the transitions between developmental stages and specifies floral organ identity			[81]
	AP2	*N. tabacum*				[90]
	AGOs	*A. thaliana*				[94]
	AP2	*H. spontaneum*				[88]
	NNC1	*G. max*				[106]
	MYBs	*S. lycopersicum*				[107]
miR319	TCPs	*A. thaliana*	Cooperatively regulates downstream genes, such as *CUC* genes, for cotyledon boundary, leaf serration formation, and other physiological responses.			[45]
	MTR_3g011610	*M. truncatula*				[108]
	PvPCF5	*A.s thaliana*				[109]
miR390	ARFs	*Populus spp.*	Directs the production of tasiRNAs from *Trans-acting siRNA3 (TAS3)* transcripts to regulated *ARF* genes			[110]
	TAS	*Helianthus tuberosus*				[111]
miR393	F-box	*A. thaliana*	Regulates the expression of different sets of *TAAR* genes following pathogen infection or nitrate treatment and regulates expression of the *TIR1/AFB2* auxin receptor clade and auxin-related development			[45]
	F-box	*G. raimondii*				[81]
	AFB2	*H. spontaneum*				[88]
	AsTIR1	*Agrostis stolonifera*				[64]
miR394	F-box	*G. raimondii*	Participates in the regulation of plant development and stress responses			[81]
	F-box	*A. thaliana*				[45]
	F-box	*G. max*				[105]
miR395	Unknown	*S. lycopersicum*	An important regulator involved in sulfate transport and assimilation and a high-affinity sulfate transporter			[86]
	ATP sulfurylase	*P. virgatum*				[83]
miR396	Unknown	*G. raimondii*	Control cell proliferation, margin, and vein pattern formation			[81]
	GRFs	*A. thaliana*				[45]
	GRFs	*N. tabacum*				[90]
	GRFs	*P. virgatum*				[83]
	bHLH74	*R. sativus*				[82]
	GRFs	*A. stolonifera*				[112]
	GRFs	*A. thaliana*				[113]
miR397	LACs	*S. linnaeanum*	Functioning in lignin synthesis and are involved in the development of plants under various conditions			[45]
	cDNA l-ascorbate oxidase precursor	*P. virgatum*				[83]
miR398	Cu/Zn Superoxide dismutase	*A. thaliana*	Regulates plant responses to oxidative stress, water deficit, salt stress, abscisic acid stress, ultraviolet stress, copper and phosphate deficiency			[45]
miR399	ATP-dependent RNA helicase	*M. truncatula*	Regulates phosphate homeostasis			[108]
	ATP-dependent RNA helicase	*T. aestivum*				[93]
miR402	DEMETER-LIKE protein 3	*A. thaliana*	Regulator of seed germination and seedling growth			[109]
miR408	DEAD-box helicases	*O. sativa*	Provide an important cross-link between plant growth, development, and stress response.			[114]
	SnRK2	*T. aestivum*				[69]
	Cu-binding proteins	*N. benthamiana*				[66]
miR414	GhFSD1	*A. thaliana*	Critical role in regulating the growth and development of plants’ cell development and cell differentiation			[115]
miR474	PPR	*Populus cathayana*	Plant nutrient homeostasis			[116]
miR482	TIR-NBS-LRR	*P. trichocarpa*	Regulates defense mechanisms			[117]
	GRAS	*S. lycopersicum*				[107]
miR530	F-box	*P. trichocarpa*	Plant resistance against multiple pathogens and nutrient homeostasis			[117]
miR1444	POPTR_0001s39950	*P. trichocarpa*	Regulates copper homeostasis			[117]
miR1445	Unknown	*P. trichocarpa*	Unknown			[117]
miR1446	GRM-like protein	*P. euphratica*	Nutrient homeostasis			[84]
miR1447	ABC transport protein	*P. euphratica*	Abiotic stress tolerance			[84]
miR1448	unknown	*P. euphratica*	Disease resistance against fungal pathogens			[84]
miR1507	NBS-LRR	*G. max*	Activators of plant defense			[105]
miR1711	unknown	*P. trichocarpa*	Unknown			[117]
miR2118	APS-reductase	*Phaseolus vulgaris*	Involved in the production of 21-nt phasiRNAs			[118,119]
miRNVL5	GhCHR	*G. hirsutum*	Vital in plant response to salinity			[120]

*Squamosa promoter-binding protein-like (SPB-Like); Auxin response factors (ARFs); Dicer-like (DCL); Argonaute (AGO); Nucleotide-binding site–leucine-rich repeat (NBS-LRR); ATP-binding cassette (ABC); Gibberellin response modulator (GRM); Toll/interleukin receptor (TIR); Pentatricopeptide repeat (PPR); SNF1-related protein kinase 2 (SnRK2); Auxin signaling F-BOX 2 (AFB2); Teosinte branched1-cycloidea-proliferating cell factor (TCP); Apetala 2, (AP2); NACs (NAM, no apical meristem, petunia, ATAF1–2, Arabidopsis thaliana activating factor, and CUC2, cup-shaped cotyledon); Homeodomain-leucine zipper (HD-Zip); Squamosa promoter-binding-like (SPL).*

miR393 is a conserved miRNA found in several plant species. The rice miR393 family consists of two members: osa-miR393 and osa-miR393b [62]. The expression of osa-miR393 significantly changed under salt and alkaline stress, whereas the expression of osa-miR393b remained stable. According to the authors, over-expression of athmiR395c detained seed germination in Arabidopsis under conditions of excessive salt or dehydration, but over-expression of athmiR395e improved seed germination under saline conditions [121]. As a result, miRNAs from the same family may play a variety of roles. Next-generation sequencing (NGS) techniques were used to explore soybean stress-related miRNAs [61,122]. The researchers discovered that under salt stress circumstances, soybeans generated 133 conserved miRNAs from 95 distinct miRNA families, differentially expressing 50 miRNAs [122]. miR159 and miR319 expression increased in artichoke tissues following a saline solution treatment [123]. *Medicago truncatula* and *M. sativa* had different expression patterns for several miRNAs, including miR156 and miR166, indicating that these two plant species had different levels of salt tolerance [124]. According to DS of transcripts and miRNAs, salt stress inhibited the expression of most miRNAs in banana roots. Moreover, other stress-related functions, such as cellular homeostasis, metabolism, and cellular stress responses, were also inhibited [125]. Salt-stress-sensitive miRNAs have been identified in nursery seedlings of *Eutrema salsugineum* using Solexa sequencing, which was vital to analyzing the direct and indirect responses to salinity [126]. The authors proposed that salt-responsive (SR) precursor miRNA genes contain numerous stress- and phytohormone-regulatory cis-regulatory elements [126].

Salt-responsive miRNAs of *Solanum lycopersicum* and *S. pimpinellifolium* were characterized by generating libraries of miRNAs from NaCl-treated and untreated seedlings. miRNAs were found to belong to 45 different families, with 95 conserved or known and 254 unknown. In response to salt stress, 109 novel and 14 conserved miRNAs were significantly regulated—specifically, the interaction of the miR156e-5p with miR23b and miR50a in *S. pimpinellifolium* [107]. In wild emmer, researchers have also identified salt-induced miRNAs. They discovered a total of 212 miRNAs, 50 of which were salinity-sensitive, with 32 significantly up-regulated and 18 down-regulated. miR172b and miR1120a, as well as miR393a, were the most significantly differentially expressed. Based on these results, wild wheat miRNAs can be explored in terms of their biological functions and evolution [127].

One-hundred-fifty conserved miRNAs and 348 new miRNAs were discovered in salt-treated *Oryza glaberrima* (African rice). Salinity stress differentially regulated 29 known and 32 new miRNAs. The GO and Kyoto encyclopedia of genes and genomes (KEGG) analyses showed that salinity stress tolerance was mediated by several targets. Based on the analysis of RT-PCR data, it appeared that some miRNAs were expressed in the adipose tissue in the same manner as indicated by Illumina sequencing data. An inverse correlation was found between the target gene and miRNA expression [128]. osa-miR396c was overexpressed in transgenic creeping bentgrass (*Agrostis stolonifera*) in perennial monocots under saline conditions. The leaves in mutants were smaller, the internodes were shorter, the leaves had fewer leaf veins, and there were fewer epidermal cells per square inch as compared with their wild counterparts. When exposed to high salinity levels, transgenic plants showed improved water retaining capacity, chlorophyll content, and cell membrane integrity. This provided insights into miRNA-mediated regulatory networks by establishing molecular links between upstream regulatory elements and downstream functional elements in the miR396 pathway [112]. The overexpression of a particular miRNA, miR1841, in rice (*Oryza sativa*) plants significantly alleviated salt stress [129]. Originally discovered in Dongxiang wild rice (*Oryza rufipogon Griff.*) [130], miR1861 plays a crucial role in salt stress in rice (*Oryza sativa*) plants.

The researchers investigated the role of 40 miRNAs belonging to 19 different families in the superfruit guava (*Psidium guajava* L.). In response to salinity stress, seven guava miRNAs (miR156f, miR160c, miR162, miR164b, miR166t, miR167a, and miR390b) showed differential regulation [33]. In another study, under salt conditions, the Hassawi-3 (a faba bean SS genotype) underwent a comparison with the ST-ILB4347 genotype. It was found that Hassawi-3 and ILB4347 differentially expressed 527 and 693 miRNAs, respectively. There was also a significant increase in 284 miRNAs in Hassawi-3 under control and a reduction in 243 miRNAs in ILB4347 plants during stress conditions [131].

The NGS and qRT-PCR validations of miRNAs associated with salinity stress responses in Niger (Guizotia abyssinica Cass.) were recently conducted [132]. The research findings identified 212 conserved miRNAs in oil-producing plants (300 mM NaCl) and 203 miRNAs in control libraries. From these libraries, 6 and 16 new miRNAs were predicted from stressed and control Niger, respectively. Based on qRT-PCR evaluations, it was found that six miRNAs were up-regulated (miR166, miR169, miR156, miR6173, miR6478, and miR166), while four miRNAs were down-regulated (miR166e, miR156a, miR159b, and miR169h) [132]. Setaria viridis, which is an emerging monocotyledonous grass model species, has similarly been shown to accumulate miR397 from Arabidopsis transformant lines [133]. A range of developmental phenotypes was observed in the transformed lines, termed Sv-MIR397 plants, exhibiting mild to severe dwarfism. By using qRT-PCR, the authors determined that miR397 overabundance repressed expression of the LAC target gene and reduced lignin contents in the Sv-MIR397 transformant plant population. Sv-MIR397 transformants were also more sensitive to salt stress than WT Arabidopsis plants were after exposure to a seven-day salt stress regime [133].

Different cultivars of the same plant species may exhibit variable responses to salt stress conditions resulting in differential expression of miRNAs. Among two rice cultivars, IR26 (sub. Xian) and JCQ and Jiucaiqing (sub. Geng), 73 mature SR-miRNAs were identified through DS. In addition to transcriptional regulation, miRNAs targeting these genes were also involved in responding to stimuli [67]. Moreover, to determine whether alfalfa and WT plants have different SR-miRNAs, researchers constructed sRNA libraries. Alfalfa plants grown under normal or saline conditions contained 128 miRNAs. Accordingly, 29 and 23 different miRNAs were differentially expressed between the Alfalfa-CK and WT-CK salt-supplemented plants, respectively [134]. Correspondingly, SR-miRNAs in sweet potatoes were uncovered through DS using libraries constructed from leaves and roots of sweet potatoes treated with NaCl (Na-150) and NaCl-free (CK) [135]. The results exhibited the existence of 175 novel and 66 conserved miRNAs. Salinity stress increased 51 miRNAs (22 known and 29 novel miRNAs) and significantly reduced 76 (61 known and 15 novel miRNAs) in sweet potato leaves. In roots, 13 miRNAs were significantly up-regulated (12 known and 1 novel miRNA), and 9 were significantly down-regulated (seven known miRNAs and two novel miRNAs) [135]. Furthermore, miRNA profiling showed that miR169, miR395, miR396, miR397, miR398, and miR408 played major roles in shoot and root tissue of two S. viridis accessions (A10 and ME-034V) when exposed to salinity [136]. The sRNA and DgS of barley, which is among the most salt-tolerant cereal crops, revealed 40 and 51 SR-miRNAs in the roots and shoots of ST-XZ16 and Golden Promise (GP), respectively [88]. There were several miRNAs involved in salt tolerance in roots, such as miR156d, miR164a, miR393a, miR319a, and miR172b, which targeted Uridine 5′-diphospho-glucuronosyltransferase (UDP-glucuronosyltransferase, UGTs), NACs (NAM no apical meristem, petunia, ATAF1–2 Arabidopsis thaliana activating factor, and CUC2 cup-shaped cotyledon), TIR1, TCP4, and APETALA2 (AP2). It has been suggested that miR159a, miR169i, miR319a/miR396e modules, and miR172b, which regulate MYB33, TCP4, growth regulating factors (GRFs), and AP2, contributed to salt tolerance in shoots [88].

Using Illumina high throughput sequencing and comprehensive in-silico analysis to obtain insight into salinity tolerance in the roots of two contrasting wheat cvv, namely Suntop (ST) and Sunmate (SS), 110 conserved and 81 novel miRNAs were identified. There were 191 miRNAs identified in both cultivars. Among them, 181 miRNAs were shared between the two cultivars. In total, these miRNAs belonged to 35 known families, of which 23 were conserved, and 12 were unique families. Saline conditions induced 43 and 75 miRNAs in Suntop and Sunmate, respectively [137]. These findings improved our understanding of how miRNAs participate in the cellular process of salt tolerance, and this knowledge may assist in genetically improving wheat cultivars. SR-miRNAs are being discovered in great numbers as genomic technology and procedures have evolved, leading toward our better understanding of their targets and gene expression.

## 4. The Target Genes and Related Pathways of Salt-Responsive miRNAs

Multiple miRNAs that target genes from the same family are frequently found in various plant species. Evidence suggests that miRNAs can selectively operate on certain target genes in a variety of contexts [138]. Based on maize miRNA sequencing, TFs are often targeted by miRNAs that are critical for the growth of plants and the formation of their organs. Earlier model plant research confirms these results. Targets of zma-miR164a/b/c/d are known to be *MYBs*, *NAC1*, and *HD-ZIP (Homeodomain-leucine zipper proteins)* [98].

Salt stress responses in plants involve several regulatory proteins, including *MADS-box* and *zinc-finger proteins* [139], that can be miRNA targets. In addition to TFs, various metabolic pathways and physiological functions are regulated by miRNA-targeted genes. The genes encoding salt-stress-responsive enzymes and proteins in plants include cytochrome oxidase and NADP-dependent malic enzyme. Both are predicted miRNA targets [140]. For example, in salt-induced soybeans treated with sulfate deficiency, miR395 regulates the sulfurylase and *ASP1* genes. Moreover, miR395 is important in energy supply maintenance [141,142]. In saline conditions, miR394a, which regulates the *F-box* proteins, was drastically up-regulated in the leaves and roots of *P. euphratica* [143]. A similar trend was seen in Arabidopsis ath-miR394 during saline conditions [53]. However, a negative expression pattern was observed in rice cultivar IR26 for osa-miR394:*LOC_Os01g6940* [67].

It has been found that genes from the *LAC* family, which are implicated in salinity response [144], are homologous to cca-miR397 and cca-miR399. With Arabidopsis as a heterologous system, *S. viridis* miR397 could reduce lignin content and increase salt stress sensitivity by repressing three Arabidopsis LAC genes. Furthermore, in salt-stressed *Col-0*, *MIM399*, and *MIR399* plants with an elevated miR399 abundance and altered expression of the *PHOSPHATE2 (PHO2)* target genes, significant changes were observed in the expression levels of the *PO4* transporter genes (*PHT1;4* and *PHT1;9. PHT1;4* and *PHT1;9*). *PO4* transporters were elevated in salt-stressed Arabidopsis and could enhance *PO4* translocation from the roots to the shoots, which would increase the amount of this precious cellular resource available. To maintain essential biological processes or to mount an adaptive response to salt stress, Arabidopsis aerial tissues could use salt stress to maintain essential biological processes [145]. In this model, it was clearly demonstrated that the anthocyanin biosynthesis pathway was accelerated to a higher degree when induced by stress in the *MIR399* transformant lines compared to the degree to which this antioxidant pigment production pathway was stimulated in *Col-0* plants or *MIM399* plants [145]. Using the above molecular manipulation model, the same authors’ group showed that Arabidopsis responded to salt stress by altering miR396/*GRF* expression [113].

B3 DNA-binding domain proteins and *auxin response factors (ARFs)* are bound by miR160 [146]. Growth, development, and response to environmental conditions are influenced by several *ARFs* [147,148]. In RNA-seq analyses, 29 and 30 SR-*ARF* members from *A. duranensis* and *A. ipaensis* were identified, respectively [149]. There was a prediction that miR160 might target *Arahy.7DXUOK*, an *ARF* gene. Plants transgenic with miR160OX may exhibit increased salt tolerance due to overexpression of miR160. The *TATA-box* is essential for the expression of *ARF* genes, as most members of the *ARF* genes participate in related signaling pathways. *P-box* elements [150], tricarboxylic acid (TCA) elements [151], *ABRE (ABA-responsive elements)* motifs [152], Gibberellic acid responsive elements (GARE) motifs [153], *TGACG* and *CGTCA* motifs have all been identified as hormone-responsive elements in *ARF* gene members. The two wild peanuts showed functional diversity and involvement in various biological processes, including responses to phytohormones, abiotic stresses, and even tissue development, according to a cis-element analysis [149].

Based on GO analysis, many important physiological players (genes) have recently been targeted by SR-miRNAs, such as *ARFs*, AA/IAA-*ARF*-dimerization, Cytochrome P450, *Chlorophyll A-B* binding protein, NADPH-cytochrome P450 reductase, *Homeobox leucine-zipper proteins*, *NF-YA*, and *MYBs* [132]. miR166a is a prominent SR-miRNA that is associated with an increased rooting rate, DNA glycosylase, and phosphotransferase activity in *Larix leptolepis*, promoting lateral root formation and carbohydrate metabolism [154]. This particular miRNA targets *HD-Zipsi* which is essential for root development in Niger plants during salt stress [132].

**Figure 1:**
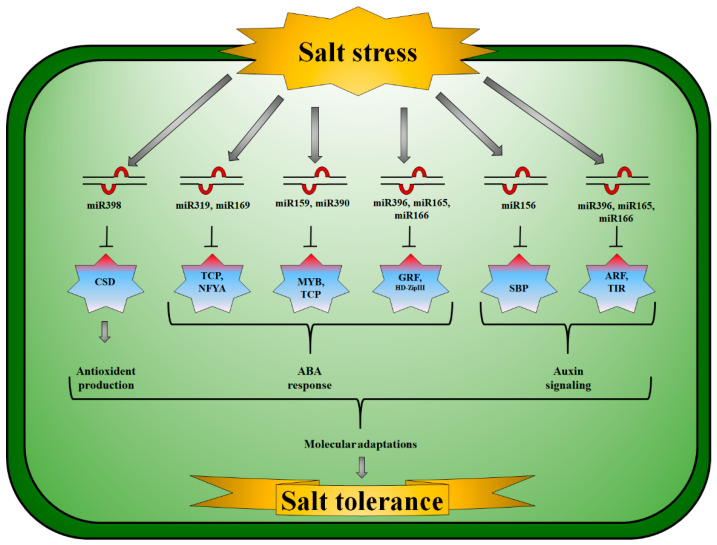
Salt-responsive microRNA and their corresponding targets and signaling pathways in plants. miR398/*CSD (Copper superoxide dismutase)* module produces antioxidants, miR319/*TCPs (Teosinte branched1/Cycloidea/Proliferating cell factor)*, miR169/*NFYA (Nuclear factor Y subunit A)*, miR159/*MYBs (Myeloblastosis)*, miR390/*TCPs*, miR165/miR166/*HD-Zip (Homeodomain-leucine zipper proteins)*, and miR396/*GRF (Growth-regulating factors)* module are key ABA-responsive elements. miR156/*SBPs (Squamosa promoter binding protein)*, miR165/miR166/*TIR (Toll/Interleukin receptor)*, and miR396/*ARF (Auxin response factors)* modules play vital roles in auxin signaling to initiate molecular response to tolerate salinity in different plant species.

Auxins (AA) are vital hormones in the development of lateral roots and apical dominance in plants by influencing cell division, elongation, and differentiation [155]. In *Vigna unguiculata*, miR160a expression increased in saline conditions. miR160a and miR160b expression, on the other hand, increased after five hours of salt treatment but declined 24 h later [104]. The target gene for miR398b encodes *Cu/Zn-SOD*; however, miR398b and miR395 are generally expressed in opposite directions [156] (Figure 1). *APS* and *Kelch* motif proteins are molecular targets of miRNAs. Plants produce ATP using *APS* and the pyrophosphate anion (Ul96) [157]. Plant development was considerably impacted by miR396 inhibition via targeting *GRFs* [158] (Figure 1). Specifically targeting the *cytochrome oxidase subunit I* gene, the *Z. mays* miR396 family was down-regulated in plants growing in saline soil [102]. *P. cathayana* had a diminished expression of miR396f and activated target genes that produce *GRF* when salt stress was applied [116].

*AGO1* gene, which encodes the enzyme that creates RNA from miRNAs, is controlled by miR168 [159]. miR168 and *AGO1* affect miRNA target genes in the same manner. Salt-stressed maize also produces miR168, which performs a regulatory function [101] miR161 and miR173 expression increased, while pri-miR161 and pri-miR173 expression decreased [94]. In the cytoplasm, these miRNAs stabilized, whereas their expression in the nucleus was negatively regulated by *AGO1*. It was concluded that *AGO1* took part in interacting with chromatin at the loci of the above miRNAs, causing the transcriptional complex to disassemble and short, unpolyadenylated transcripts to be released [160].

Target genes are implicated in a range of critical pathways, including AA and ethylene (ET) signaling, RNA-mediated silencing, and DNA methylation [161]. According to researchers, miRNVL5 and *GhCHR* are two critical components involved in salinity response. Further, salt stress increased the expression of GhCHR (a gene of miRNA ovary line 5) while reducing the expression of corresponding miRNA [120]. It was concluded that the constitutive expression of miRNVL5 made transgenic plants susceptible to salt stress, whereas GhCHR expression enhanced salt tolerance [162]. In NaCl-treated and untreated seedlings of *S. lycopersicum* and *S. pimpinellifolium*, SR-miRNA’s target genes were predicted via GO and miRNA involvement in salt-stress-related biochemical pathways, including photosynthetic pathways, hormonal signaling pathways, phospholipid signaling pathways, and calcium signaling pathways [107]. Through the use of GO and KEGG analyses, targets for miR172b and miR1120a were predicted in wild emmer. Target proteins such as TFs and stress-related proteins were enriched in salinity-responsive miRNAs [127]. The GO classification and KEGG pathway analysis of the potential target genes in WT and M. alfalfa plants under normal and saline conditions predicted that the majority of target genes were related to plant growth and development and showed significant differences between WT and M. alfalfa plants. Moreover, miR172-*CNGC (cyclic nucleotide-gated channel)*, miR319-*CAX2 (CATION EXCHANGER 2)*, miR408-*NHX (NA^+^/H^+^ exchanger)*, and miR2590-*CHX14/15 (cation/H^+^ exchanger)* were significantly up-regulated in M. alfalfa plants compared with WT plants, suggesting that M. alfalfa plants have higher ion transport levels [134].

As specified by GO and KEGG, SR-*O. glaberrima* miRNA targets several genes that participate in salinity stress resistance pathways [128]. Four potential miR396 targets were additionally identified in creeping bentgrass, and the levels of these targets were elevated when the plants were stressed by salt. In stressful conditions, miR396 was found to be required for salt stress tolerance through both functional and regulatory proteins (TFs and protein kinases) [112]. Based on results from the psRNATarget tool, *P. guajava* miRNA target transcripts were characterized to have 49 potential targets, mostly involved in metabolism, cellular development, and stress responses [33]. Faba bean genotypes Hassawi-3 (SS) and ST-ILB4347 have specific miRNA targets involved in regulating specific SR genes, primarily TFs, *LACs*, *SODs*, *plantacyanins*, and *F-box* proteins.

Salinity-induced miRNAs and their targets are involved in corresponding biological networks and associated pathways such as ABC transportation, MAPK (mitogen-activated protein kinase) signaling networks, and plant hormone networks, indicating that miRNAs play a role in salt stress tolerance in the ILB4347 genotype [131]. Similarly, in-silico analysis of two contrasting SR-wheat cultivars (Suntop and Sunmate) identified more than 800 targets for the 75 known miRNAs. Signaling activities of miR156, miR160, miR171, miR319, miR159, miR9657, and miR59 were linked to *ARFs*, *SPLs (Squamosa-promoter binding protein-like)*, and *Scarecrow-like 6 (SCL6)*. It was predicted that the proteins *PCF5 (binds to the core sequence of the promoter)*, *R2R3-MYB*, and *CBL-CIPK (CBL-interacting protein kinases)* were involved in salt tolerance [137]. Similar to this, a miRNA-DgS of sweet potato samples treated with and without salt revealed that the *SPL*/miR156 and miR169/TOP1 modules, as well as *GC4*, *HSP90*, *UBXN1*, miR393/*AFB2*, and miR162/*DCL* modules, played vital roles during saline conditions [135].

miRNAs have been found to regulate hormone pathways in plants under salt stress. Plant stress responses may be linked to AA signaling through miR160, miR167, and miR393 [163]. As a result of their low expression levels, miR393, miR160, and miR167 are slightly inhibited by the expression of ARFs under non-stressed conditions. Up-regulated miR393 repressed AA signaling by lowering *TIR1*, which increased AA/IAA-ARF heterodimerization under stress conditions [164]. *ARF* levels were also directly reduced by miR160 and miR167 up-regulation [164] and miR162 down-regulation [32]. Salt treatment of Kentucky bluegrass (*Poa pratensis* L.) led to changes in the expression levels of miRNAs [165]. Salt treatment increased the expression of miR162, miR173, miR391, miR408, miR773, and miR857 by 70%, then declined to levels similar to those of the control after 24 h. A 20% decrease in miR775 and miR827 expression levels was observed after 24 h, followed by an 80% decrease after 144 h. miR841 expression increased by 50% after 24 h of salt treatment but stabilized after 144 h. Salt treatment significantly increased the expression of *ARFs* between 12 and 144 h, respectively. When salinity stress was applied to the callus, miRNAs were found to regulate SR-gene families [165].

Several studies have established that miRNAs target the *TIR1* or *ARF* genes to contribute to salt stress responses [64,166]. He et al. [110] found that salt-induced miR390 expression stimulated tasiARF production for the degradation of *ARF4* transcripts that influence ARFs. Inhibition of salt-induced AA signaling was facilitated by the decreased expression of *ARF4*. Nodulation and salt stress are regulated by miR390 in dual ways. By overexpressing miR390 in *M. truncatula*, lateral root growth is stimulated. Nodule organogenesis and rhizobial infection are prevented, and nodulation genes are inhibited, while miR390/TAS3 (trans-acting-small interfering RNA3) inactivation leads to more nodulation and rhizobial infections [167]. Using NaCl concentrations of 100 mM and 300 mM, the authors found that in *Helianthus tuberosus*, miR390 expression is induced by 100 mM, whereas miR390 expression is inhibited by 300 mM [111]. A miR390-TAS3-tasiARFs module pathway plays distinct functions in regulating salt stress, and nodulation is also complex. Furthermore, a recent study revealed that miR167 genes were constitutively diminished during organogenesis under target-mimicry-based conditions [166]. Under stress, miR167 mimic lines exhibited a greater magnitude of organogenesis compared to the parent line (cultured in NaCl concentrations of 12.5 and 25 mM). As a consequence of miR167 reduction paired with salt stress (up to 12.5 mM), AA transporter genes *AUX1 (Auxin influx transporter 1)*, *PIN1 (Peptidylprolyl Cis/Trans Isomerase*, *NIMA-Interacting 1)*, and *PIN2* showed synergistic effects resulting in enhanced callogenesis and reduced organogenesis. miR167 reduction-associated signaling pathways were reflected in the increased relative water content, chlorophyll, and antioxidant activity of in-vitro grown miR167 mimic shoot initials [166].

Additionally, miRNAs modulate the ABA and ET metabolic pathways to provide resistance to salt toxicity. There are several miRNAs involved in the ABA metabolic pathway during salt stress. i.e., miR156, miR172, miR393, miR394, and miR399 [168,169,170,171,172]. Moreover, salt stress regulates miR319 and the ET metabolism pathway [173]. ABA-mediated pathways are negatively regulated by *scaffold protein receptors for activated C kinase 1 (RACK1)* [174], e.g., in *A. thaliana* through miR393s [175]. Several studies have demonstrated that miR6478 controls the ET signaling pathway in Niger plants under salt stress, targeting *AGO/DCL protein*, *PAZ (Piwi*, *Ago*, and *Zwille)*, and *CTR1 (CONSTITUTIVE TRIPLE RESPONSE 1)*, thus promoting miRNA turnover [132]. There is evidence that the *CTR1* gene participates in the ET signal transduction pathway. Plant ET responses are negatively regulated by the amino terminus of *CTR1*, which was previously reported to form a complex with ET [176]. Furthermore, *OsNAC2* overexpressing lines (*ZUOErN3* and *ZUOErN4*) were more salt tolerant than WT rice seeds since their levels of ABA were higher in comparison with WT seeds [177]. RT-PCR revealed that *OsNAC2*-overexpressing plants expressed significantly more ABA biosynthesis genes *OsNCED1 (9-cis-epoxycarotenoid dioxygenases)* and *OsNCED3*, as well as higher levels of expression of stress-responsive genes *OsP5CS1 (pyrroline-5-carboxylate synthase 1)*, *OsLEA3 (late embryogenesis abundant)*, and *OsRab16 (responsive to ABA)* [177].

ROS accumulates in plants as a result of oxidative stress during salt stress conditions [178]. *SOD* converts superoxide radicals into molecular oxygen and hydrogen peroxide, which provide the first cellular defense against oxidative stress. By introducing miR397, miR398, miR408, and miR528 in the cell, *Cu/Zn-SOD*, and *LACs* are suppressed, thereby controlling ROS accumulation and the availability of Cu ions. Additionally, miR398, miR408, and miR528 are involved in heavy metal transport regulation [26]. Through its effects on iron *SOD (Fe-SOD, FSD)* in cotton, miRNA414c affects salinity tolerance [115]. *L-ascorbate oxidase (LAO)* is an enzyme that breaks down ascorbate, a molecule that detoxifies H_2_O_2_. miR12477 plays a vital role in the inhibition of LAO. Salt stress results in low ROS accumulation when miR12477 expression is high [34]. The *AP2/ERF (Ethylene response factors)* domain-containing TF gene *INDETERMINATE SPIKELET1 (IDS1)* plays a crucial role in interacting with ROS produced by salt-treated plants via miR172a/b [179].

## 5. Conclusions and Future Prospects

Gene expression patterns affect plants’ responses to salinity stress. miRNAs, which are active post-transcriptional regulators, are known to regulate stress-related genes. Understanding how miRNAs affect genetic expressions will enable us to understand miRNA’s role in saline conditions in many plant species. The new availability of whole plant genomes and high-throughput sequencing technology has fueled research into miRNA control under salt stress. Because miRNAs are so crucial in gene regulation networks, learning more about them should help us better understand plant salt tolerance responses. Understanding miRNA-mediated regulation networks could lead to new strategies to improve plant salt tolerance genetically. Although we are still learning about miRNA evolution, studies on salinity-responsive miRNAs and their corresponding target networks in different cell types may uncover how miRNA target networks operate in these different cell types. Identifying miRNAs that change expression levels in response to salt stress in various agricultural plants and their target genes remains a slow progress. The solution reveals new elements in plant stress tolerance and helps unravel the salt stress response’s complex regulatory network. If we can better understand miRNA activity under salt stress, we may be able to use miRNA-mediated gene regulation to improve plant stress resistance, particularly for economically important crops, to ensure future food security.

## Data Availability

Not applicable.
